# *Dolichandrone serrulata* flower improves seminal biochemical parameters and proteins in T2DM rats induced by a high-fat diet and streptozotocin

**DOI:** 10.1080/13880209.2022.2124279

**Published:** 2022-10-07

**Authors:** Tarinee Sawatpanich, Chadaporn Chaimontri, Alexander Tsang-Hsien Wu, Sitthichai Iamsaard, Supataechasit Yannasithinon

**Affiliations:** a Department of Anatomy, Faculty of Medicine, Khon Kaen University, Khon Kaen, Thailand; b TMU Research Center of Cancer Translational Medicine, Taipei Medical University, Taipei, Taiwan; c The PhD Program of Translational Medicine, College of Science and Technology, Taipei Medical University, Taipei, Taiwan; d Clinical Research Center, Taipei Medical University Hospital, Taipei Medical University, Taipei, Taiwan; e Graduate Institute of Medical Sciences, National Defense Medical Center, Taipei, Taiwan; f Research Institute for Human High Performance and Health Promotion (HHP & HP), Khon Kaen University, Khon Kaen, Thailand; g Department of Pre-Clinical, Faculty of Medicine, Mahasarakham University, Mahasarakham, Thailand

**Keywords:** Seminal vesicle, caspase, type 2 diabetes

## Abstract

**Context:**

Although *Dolichandrone serrulata* (Wall. ex DC.) Seem (Bignoniaceae) flower (DSF) improves hyperglycaemia, testicular damage and sperm quality in type 2 diabetes mellitus (T2DM) animals, its effects on the seminal vesicles, secreting seminal plasma, are unknown.

**Objective:**

This study reports the protective effects of DSF on seminal dysfunction in T2DM rats.

**Materials and methods:**

Male Sprague-Dawley rats were divided into four groups (control, T2DM, T2DM + DSF200 and T2DM + DSF600; 10 animals/group). The control group was fed a low-fat diet for 14 days prior to single saline injection, whereas T2DM group was given a high-fat diet and injected with streptozocin (50 mg/kg body weight). The T2DM-induced rats were fed DSF orogastrically (200 and 600 mg/kg body weight) for 28 consecutive days. At the end of the experiment, biochemical components, malondialdehyde (MDA), histology and protein expression in seminal lysate were evaluated.

**Results:**

DSF increased the levels of serum phosphorus (13.66 ± 0.59 mg/dL), ALP (11.85 ± 0.99 U/L), GOT (3938.23 ± 251.41 U/L) and GPT (34.16 ± 4.93), decreased MDA levels in seminal tissue, and elevated the serum testosterone in the T2DM rats. Treatment with DSF ameliorated histological damage, significantly increased seminal 44 and 31 kDa TyrPho protein expression, and decreased that of caspase 3 and 9.

**Conclusions:**

DSF extract was able to mitigate seminal dysfunction in T2DM rats via improvements of tyrosine phosphorylation, testosterone level and biochemical substances, as well as reductions of caspase proteins. DSF may be developed as an alternative medicine in treating of T2DM male subfertility and progressive complications.

## Introduction

Diabetes mellitus (DM) is currently a global public health crisis, with case numbers rapidly increasing annually. Type 2 DM (T2DM) can lead to various disorders and complications including sexual dysfunction and neuropathy (Ali et al. 1993; Faselis et al. 2020). In particular, neuropathy has been shown to cause sexual impairment and infertility due to dysfunction of seminal vesicle secretion (Ali et al. 1993). A previous study reported that infertile DM patients with neuropathy may exhibit erectile dysfunction and changes in seminal plasma content (La Vignera et al. 2013). This impairment to seminal secretion has been known to cause male infertility (Ali et al. 1993; La Vignera et al. 2011).

The seminal vesicles are important accessory sex glands that produce a complex milky fluid containing proteins, enzymes, macro- and microelements, lipids, and essential nutrients. Additionally, seminal fluid is composed of various metabolic substances including calcium (Ca), phosphorus (P), fructosamine (FRA), magnesium (MG), glutamic oxaloacetic transaminase (GOT), glutamic pyruvic transaminase (GPT) and alkaline phosphatase (ALP), which are important for sperm viability, motility and acrosome reaction (Mahamud et al. 2015; Talluri et al. 2017). Such substances can be used as potential biomarkers for estimating seminal quality (Pesch et al. 2006; Tvrda et al. 2013; Macanovic et al. 2015), and the seminal proteomic profiles in fertile and infertile men have been found to differ considerably (Cadavid et al. 2014). Changes in many tyrosine-phosphorylated (TyrPho) proteins have recently been found in damaged reproductive tissue of infertile animal models (Sukhorum and Iamsaard 2017; Tongpan et al. 2019; Tangsrisakda and Iamsaard 2020; Arun et al. 2021). Such alterations have been reported in the testes and seminal vesicle of hyperglycaemic animals (Sampannang et al. 2017, 2018, 2019; Yannasithinon et al. 2021). TyrPho proteins play an essential role in sperm capacitation and acrosome reaction (Said et al. 2004; Kumar 2007). Moreover, increases in TyrPho proteins and caspases (3 and 9) have been found in the seminal vesicle tissue and fluid of stress animals (Iamsaard et al. 2021). Caspase proteins are particularly involved in the caspase mechanism of various andrological disorders (Said et al. 2004; Kumar 2007). It has thus been proposed that changes of seminal plasma could indicate male infertility (Naz and Rajesh 2004; Said et al. 2004; Tsounapi et al. 2017; Yannasithinon and Iamsaard 2019; Yannasithinon et al. 2021).

*Dolichandrone serrulata* (Wall. ex DC.) Seem (Bignoniaceae) flower (DSF) has been used to reduce the blood glucose levels in some DM patients. Recently, a DSF extract was found to alleviate high fasting blood glucose (FBG) and testicular malondialdehyde (MDA) and to increase the serum testosterone levels of T2DM-treated animals (Yannasithinon et al. 2021), possibly as a result of its antioxidant capacity, phenolic compounds and terpenoids (phytoandrogens; rengyolone and cleroindicin B) (Chaimontri et al. 2021). Additionally, DSF has been shown to be abundant in antioxidants, have oxidative-scavenging ability, and exhibit antibacterial properties (Thummajitasakul et al. 2014; Phanthong et al. 2015). Moreover, a previous study demonstrated that DSF could decrease the testicular MDA level (Yannasithinon et al. 2021). Despite this, the effects of DSF extract on seminal vesicle structure and function in T2DM animal model have yet to be demonstrated, as has the association between MDA and apoptotic markers such as caspase 3 and 9. This study was thus conducted to investigate any alterations to biochemical components, histology, or potential protein expressions in the seminal vesicles of T2DM rats after administration with DSF extract.

## Materials and methods

### Animals and ethical considerations

Forty adult male Sprague-Dawley rats aged 6–8 weeks were purchased from Nomura Siam International Co., Ltd. (Bangkok, Thailand). All animals were maintained under controlled environmental conditions (23 ± 2 °C, 50 ± 10% humidity, 12 h light/dark cycle) at the Northeast Laboratory Animal Centre (Khon Kaen University, Khon Kaen, Thailand). The study protocol was approved by the Institutional Animal Care and Use Committee under the National Research Council of Khon Kaen University (IACUC-KKU-94/62).

### Authentication and extraction

*Dolichandrone serrulata* flower extract was prepared as described by Chaimontri et al. (2021). DSF were collected from the Ban Thum Subdistrict, Mueang District, Khon Kaen Province, Thailand (March to June 2019). The plant species was identified by Dr. Pimwadee Pornpongrungrueng, a botanist of the university’s Applied Taxonomic Research Center, and specimen vouchers were kept in the Khon Kaen University Faculty of Science Herbarium (voucher number: S. Iamsaard 02). The extraction process was as follows: the DSFs were air-dried, chopped and immersed in distilled water (1:3 ratio). Then, the DSF solution was gently boiled at 90–95 °C for 40 min. After filtration with a nylon cloth, the solution was dispersed using a Buchi R100 rotary evaporator (Cerntek Co. Ltd., Bangkok, Thailand). The surface area of the concentrated DSF extract was evaluated using cool 95% ethanol in a Scan Vac machine (SHC4000 Chemoscience Co. Ltd., Bangkok, Thailand) before lyophilization (Labconco Corporation, Kansas, MO). The DSF fraction used in this study contained total phenolic compounds, flavonoid content and antioxidant capacity with possessing of rengyolone and cleroindicin B revealed by nuclear magnetic resonance (NMR) spectrometer analyses.

### Induction of type 2 diabetes mellitus and administration

All animals were randomly allocated to four groups (10 animals/group) and consecutively treated as shown in Table 1. To confirm diabetic status after induction, blood was extracted by tail prick, and FBG was evaluated using blood glucose test strips and a monitor (Accuchek Advantage II; Roche, Mannheim, Germany). The glucose levels higher than 250 mg/dL were considered to indicate T2DM. Animals were fed either a low-fat diet (LFD; 10 kcal % fat, cat. no. D12450J, Research Diet Inc., New Brunswick, NJ) in the control group or high-fat diet (HFD; 60 kcal % fat diet, cat. no. D12492 (Research Diet Inc., New Brunswick, NJ)) for 14 consecutive days. It is noted in United States Pharmacopeia (USP) that the lard (245 g) and soybean oil (25 g) are major fat compositions of the commercial HFD. Subsequently, the rats in T2DM treated groups were fasted and intraperitoneally (i.p.) injected with a single dose of streptozotocin (STZ; 50 mg/kg body weight). After T2DM induction, DSF extracts at a dose of 200 or 600 mg/kg BW (based on doses preventing testicular damages) were orally administered (p.o.) as previously explained (Yannasithinon et al. 2021). The HFD administration was continued after the STZ injection for 28 consecutive days based on previous studies (Reed et al. 2000; Srinivasan et al. 2005; Zhang et al. 2008; Yannasithinon and Iamsaard 2019).

**Table 1.** Treatments by group.

Groups	Treatments		
	Feeding	DM induction	Administration
	(14 days)	(1 day)	(28 days)
Control	LFD (10 kcal % fat, D12450J, Research Diet Inc., New Brunswick, NJ)	0.1 M citrate solution (pH ∼4.5, via i.p.)	DW (via p.o.)
T2DM	HFD (60 kcal % fat, D12492, Research Diet Inc., New Brunswick, NJ)	STZ 50 mg/kg BW diluted in 0.1 M citrate solution (pH ∼4.5, via i.p.) (Sigma-Aldrich, St. Louis, MO)	DW (via p.o.)
T2DM + DSF200	HFD (60 kcal % fat, D12492, Research Diet Inc., New Brunswick, NJ)	STZ 50 mg/kg BW diluted in 0.1 M citrate solution (pH ∼4.5, via i.p.) (Sigma-Aldrich, St. Louis, MO)	DSF 200 mg/kg BW dissolved with DW (via p.o.)
T2DM + DSF600	HFD (60 kcal % fat, D12492, Research Diet Inc., New Brunswick, NJ)	STZ 50 mg/kg BW diluted in 0.1 M citrate solution (pH ∼4.5, via i.p.) (Sigma-Aldrich, St. Louis, MO)	DSF 600 mg/kg BW dissolved with DW (via p.o.)

DSF: *D. serrulata* flower; DW: distilled water; BW: body weight; HFD: high fat diet; LFD: low-fat diet; i.p.: intraperitoneal; p.o.: orally; M: molar; T2DM: type 2 diabetes mellitus; STZ: streptozotocin; according to the description by Reed et al. (2000).

### Structural considerations

All animals were anaesthetized by i.p. injection with pentobarbital sodium (50 mg/kg) and the blood samples were collected by cardiac puncture. Each animal was immediately euthanized by the cervical dislocation upon completion of the blood collection. Then, the gross morphology of the seminal vesicles plus prostate glands was assessed. Seminal vesicles were fixed with 10% formalin buffer before being embedded in melted paraffin. Subsequently, the paraffin block was sectioned (5 µm thickness) before deparaffinization with xylene. The deparaffinized sections were gradually hydrated using alcohol and distilled water at decreasing concentrations. All tissue sections were routinely stained with haematoxylin and eosin (H&E) dyes to observe the characteristics of histological changes. The histological images were photographed (Nikon light ECLIPSE E200 microscope synchronized with a digital camera, Nikon, Minato, Japan). Histomorphometry was performed by determining the height of the seminal epithelia from its apical surface to the basement membrane using ImageJ (Version 1.50i, National Institutes of Health, Bethesda, MD) as described by Yannasithinon and Iamsaard (2019) and Yannasithinon et al. (2021).

### Examination of serum testosterone levels

The blood samples were centrifuged at 14,000 rpm at 4 °C for 25 min to separate the serum. After that, the serum samples were gathered and testosterone levels were estimated by antigen or antibody measurement based on changes to the electrochemiluminescence (ECL) signal before and after immunoreaction (Cobas e411 Analyzer, Roche Diagnostics, Indianapolis, IN) at Srinagarind Hospital’s Specimen Center (Khon Kaen University, Khon Kaen, Thailand).

### Sampling

The seminal fluid in the seminal vesicle gland was squeezed, extracted and centrifuged at 14,000 rpm at 4 °C for 25 min to isolate the supernatant. Then, the seminal vesicle tissue without fluid was homogenized using a glass grinder, sonicated and centrifuged at 14,000 rpm for 10 min. The supernatant called seminal tissue lysate was collected to further examinations.

### Measurement of biochemical levels in the seminal tissue

The seminal fluid sample from each animal was determined for the levels of biochemical components including Ca, phosphorus (P), FRA, MG and ALP by using various methods (described in Table 2) at the Diagnosis Clinical Chemistry Laboratory Unit, Srinagarind Hospital (Khon Kaen, Thailand).

**Table 2.** Methods and analyser used in biochemical determination.

Biochemical components	Method	Analyser
Calcium (Ca)	O-Cresolphthalein complexone	Cobas 8000 (c702, c502)
Phosphorus (P)	Molybdate UV	
Fructosamine (FRA)	Colorimetric	
Magnesium (MG)		
Alkaline phosphatase (ALP)	p-Nitrophenyl phosphate	
Glutamic oxaloacetic transaminase (GOT)	IFCC	
Glutamic pyruvic transaminase (GPT)		

### Assessment of malondialdehyde levels

The MDA concentration in seminal fluid or tissue lysate was measured by thiobarbituric acid reactive substance (TBARS) assay based on the procedure described by Luangaram et al. (2007). In brief, the seminal sample (0.6 mL) was sequentially combined with 10% trichloroacetic acid (0.5 mL), 5 mM/L ethylenediaminetetraacetic acid (0.5 mL), and then 8% sodium dodecyl sulphate (0.2 mL). All samples sat for 10 min at room temperature before incubation with thiobarbituric acid (0.5 mL). Then, the mixed seminal samples were boiled at 95 °C, resulting in pink-coloured solution. After centrifugation at 1600×*g* for 10 min, each supernatant was quantified at 542 nm absorbance using a microplate reader. The 1,1,3,3-tetraethoxypropane concentrations between 0 and 60 nM were plotted as a standard curve (*R*^2^=0.991). MDA level was expressed as nM MDA/mg protein.

### Seminal protein preparation and immuno-Western blot assay

The concentration of seminal protein was determined by absorbance at 280 nm using a NanoDrop Spectrophotometer (NanoDrop-1000 Spectrophotometer Manual, NanoDrop Technologies Inc., Wilmington, DE). Subsequently, the seminal lysate was subjected to 10% sodium dodecyl sulphate-polyacrylamide gel electrophoresis (SDS-PAGE). The electrophoresed proteins obtained from SDS-PAGE were placed onto a nitrocellulose membrane. After that, the proteins on the membrane were specifically proven with primary antibody at 4 °C overnight (see Table 3 for dilution). After washing, each membrane was further incubated with a secondary antibody attached with horseradish peroxidase (dilution 1:2000). Excess antigen–antibody complex on the membrane was washed. Seminal protein expression was observed using a chemiluminescent substrate reagent kit (GE Healthcare Life Sciences, Chicago, ‎IL) with the ImageQuant 400 imaging system (GE Healthcare Life Sciences, Chicago, ‎IL). The intensity of protein expression at each band was determined using ImageJ software (National Institutes of Health, Bethesda, MD). Bovine serum albumin (BSA; Millipore Co., San Jose, CA) and epidermal growth factor (EGF; Millipore Co., San Jose, CA) were used as negative and positive controls, respectively. Moreover, glyceraldehyde-3-phosephate dehydrogenase (GAPDH, Abcam, Cambridge, MA) was applied as an internal control.

**Table 3.** Antibodies used in western immunoblotting.

Antibody used	Species: antibody type	Origin	Dilution
Phosphotyrosine (4G10)	Mouse: monoclonal	Merck Millipore Co., San Jose, CA (05-321)	1:1000
Caspase 3 (E-8)	Mouse: monoclonal	Santa Cruz Biotechnology, Dallas, TX (SC-7272)	1:1000
Caspase 9 (96.1.23)	Mouse: monoclonal	Santa Cruz Biotechnology, Dallas, TX (SC-56076)	1:1000

### Statistical analysis

Data are shown as mean ± standard deviation (SD). A one-way analysis of variance was used to analyse the statistical significance of differences with SPSS (SPSS Inc., Chicago, IL). A *p* value <0.05 was considered statistically significant.

## Results

### Effects of DSF extract on the structure and weight of the seminal vesicles plus prostate glands in T2DM-induced rats

**Figure 1.**
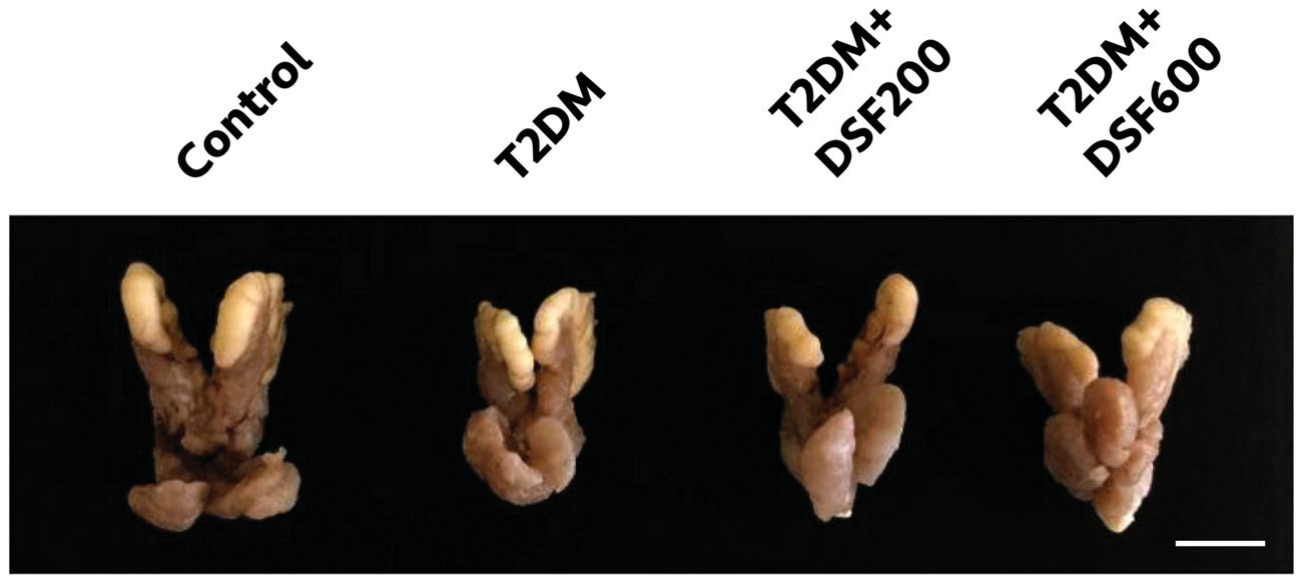
A representative photograph of the morphology of the seminal vesicles plus prostate glands of control, T2DM, T2DM + DSF200 and T2DM + DSF600 rats after treatment with DSF for 28 experimental days. DSF: *D. serrulata* flower (200 or 600 mg/kg BW); T2DM: type 2 diabetes mellitus. Scale bar 1 cm.

As shown in Figure 1 and Table 4, the seminal vesicles plus prostate glands of T2DM rats were significantly smaller and lighter than those of control animals (*p* < 0.05). After DSF administration for 28 days, both parameters were significantly higher in the treated T2DM group than in the untreated T2DM group (*p* < 0.05, Figure 1, Table 4).

**Table 4.** Absolute and relative weight of seminal vesicles plus prostate glands in control, T2DM, T2DM + DSF200 and T2DM + DSF600 groups.

	Groups			
	Control	T2DM	T2DM + DSF200	T2DM + DSF600
Absolute weight (g)	2.7216 ± 0.1555	1.6008 ± 0.3032*	2.1369 ± 0.2038**	2.1467 ± 0.2491**
Relative weight (×10^2^ g/100 g)	0.4819 ± 0.0275	0.4353 ± 0.0824	0.4949 ± 0.0472	0.5049 ± 0.0586

BGL: blood glucose levels; DSF: *D. serrulata* flower; T2DM: type 2 diabetes mellitus.

Data are expressed as mean ± SD (*n* = 10).

* Significantly different (*p* < 0.05) compared to the control group.

** Significantly different (*p* < 0.05) compared to the T2DM group.

### Effects of DSF extract on biochemical components in the seminal lysate and serum of T2DM rats

**Figure 2.**
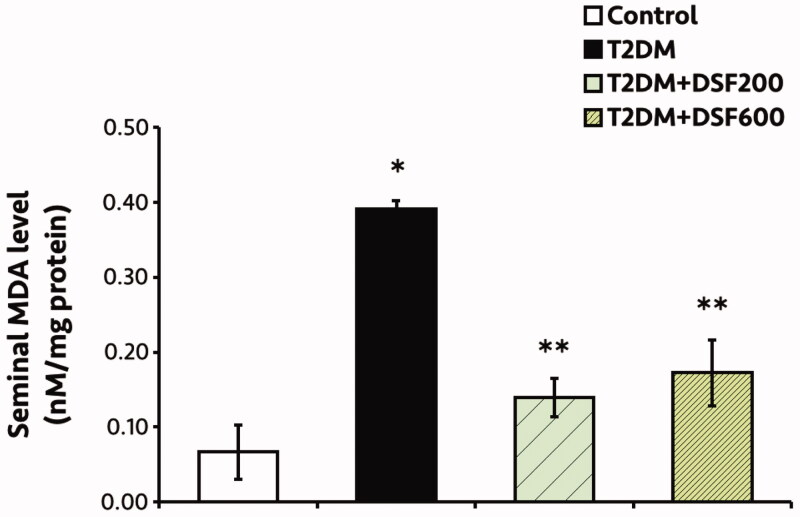
Levels of malondialdehyde (MDA) in the seminal lysate of control, T2DM, T2DM + DSF200 and T2DM + DSF600 rats. Values are expressed as mean ± standard deviation. *Significantly different (*p*< 0.05) compared to the control group. **Significantly different (*p*< 0.05) compared to the T2DM group.

Levels of Ca, GOT and GPT were significantly lower in T2DM group, while the ALP level was significantly lower in untreated T2DM rats as compared to those of control and DSF groups (*p* < 0.05, Table 5). Remarkably, DSF extract increased phosphorus (P), FRA, MG, ALP, GPT and GOT levels in the treated T2DM rats (*p* < 0.05, Table 5). Significantly, serum testosterone (T) levels were decreased in T2DM and DSF treated rats as compared to that of control group (Figure 5). It is noted that the DSF could significantly increase the T level as compared to T2DM group (*p* < 0.05, Table 5). In addition, MDA levels in the T2DM seminal tissue were significantly higher but decreased in a dose-dependent manner after treatment with DSF extract (*p* < 0.05, Figure 2).

**Table 5.** The biochemical levels in the seminal lysate and serum testosterone of control, T2DM, T2DM + DSF200 and T2DM + DSF600 rats.

	Groups			
	Control	T2DM	T2DM + DSF200	T2DM + DSF600
Ca (mg/dL)	1.28 ± 0.00	0.40 ± 0.08*	0.44 ± 0.00*	0.42 ± 0.00*
P (mg/dL)	8.27 ± 0.06	6.55 ± 1.62	13.03 ± 0.15**	13.66 ± 0.59**
FRA (mg/dL)	52.25 ± 8.48	55.14 ± 9.75	122.62 ± 3.10**	92.02 ± 7.89**
MG (mg/dL)	1.11 ± 0.00	0.75 ± 0.24	1.20 ± 0.15**	1.32 ± 0.10**
ALP (U/L)	18.42 ± 0.61	62.03 ± 14.62*	94.15 ± 3.10**	11.85 ± 0.99**
GOT (U/L)	3171.73 ± 21.80	1230.90 ± 369.60*	1488.94 ± 6.19	3938.23 ± 251.41**
GPT (U/L)	35.97 ± 0.00	12.06 ± 5.69*	14.23 ± 1.55	34.16 ± 4.93**
T (ng/mL)	6.35 ± 0.08	0.45 ± 0.00*	0.50 ± 0.02**	0.69 ± 0.01**

DSF: *D. serrulata* flower; T2DM: type 2 diabetes mellitus; Ca: calcium; P: phosphorus; FRA: fructosamine; MG: magnesium; ALP: alkaline phosphatase; GOT: glutamic oxaloacetic transaminase; GPT: glutamic pyruvic transaminase; T: serum testosterone.

Data are shown as mean ± SD (*n* = 7).

* Significantly different (*p* < 0.05) compared to the control group.

** Significantly different (*p* < 0.05) compared to the T2DM group.

### Effects of DSF extract on the histology of the seminal vesicles in T2DM-induced rats

**Figure 3.**
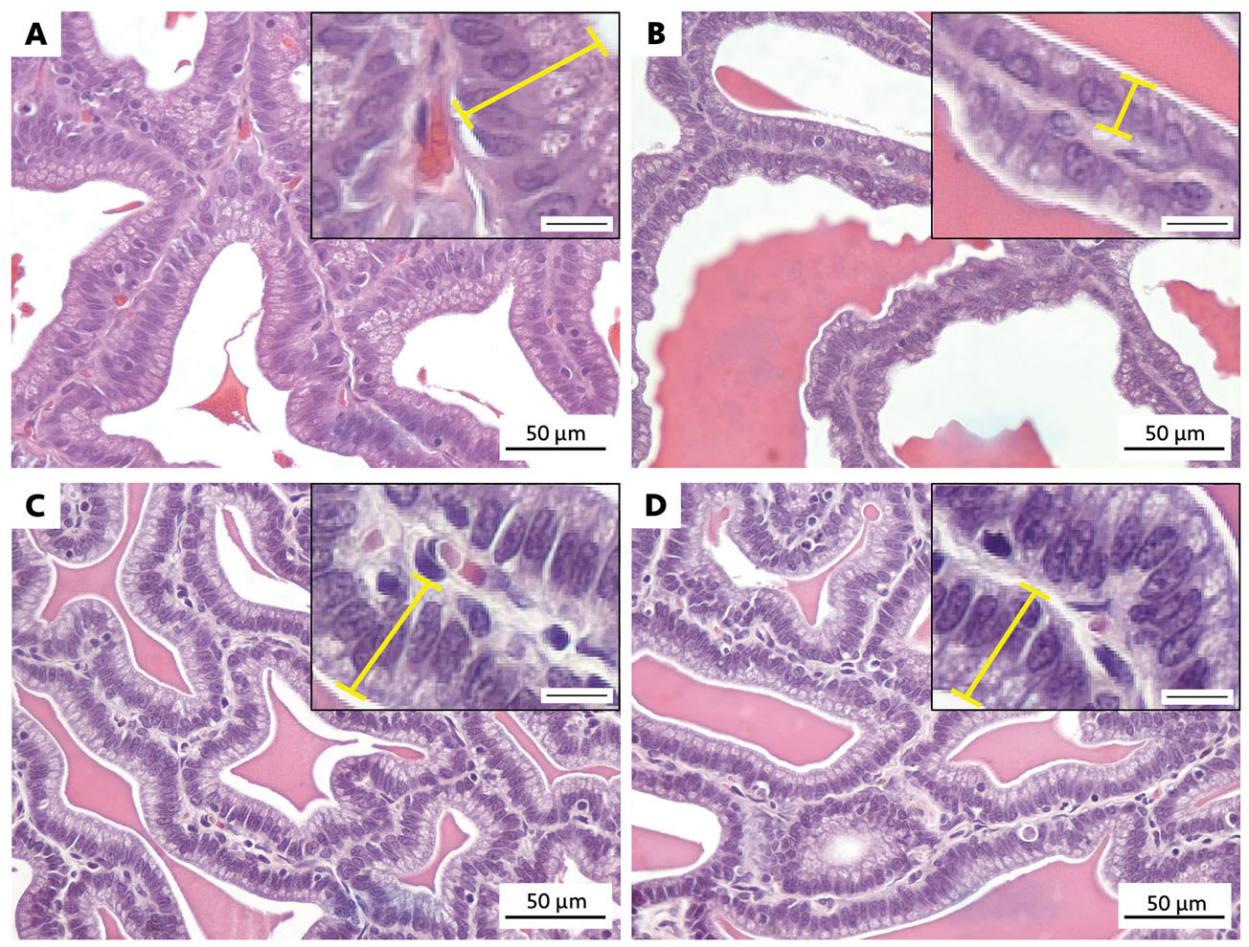
Representative histology photograph showing the seminal epithelium in control (A), T2DM (B), T2DM + DSF200 (C) and T2DM + DSF600 (D) rats after treatment with DSF for 28 experimental days. The yellow bar drawn from basement membrane to the apical surface demonstrating the high of seminal epithelial cell. DSF: *D. serrulata* flower (200 or 600 mg/kg BW); T2DM: type 2 diabetes mellitus. Scale bar (right bottom), 10 µm.

**Figure 4.**
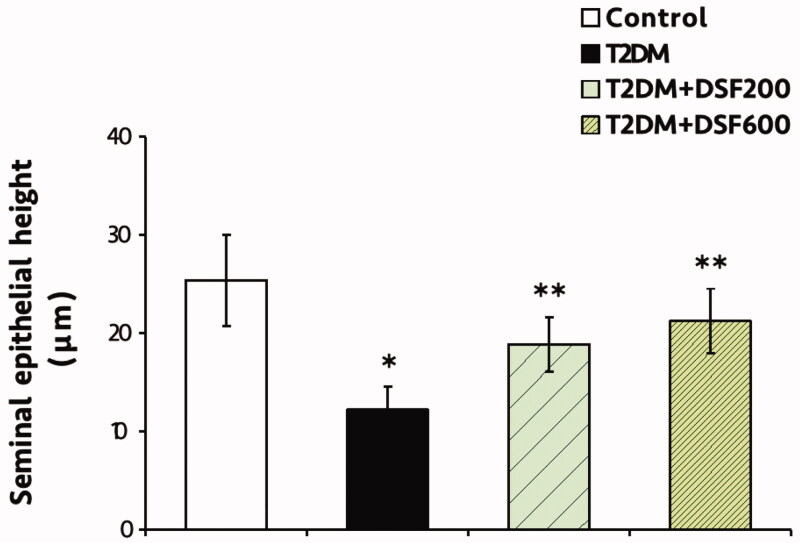
The height of the seminal epithelium in control, T2DM, T2DM + DSF200 and T2DM + DSF600 rats. Data are shown as mean ± SD. *Significantly different (*p*< 0.05) compared to the control group. **Significantly different (*p*< 0.05) compared to the T2DM group.

As shown in Figure 3, the epithelial cells observed in T2DM seminal vesicle were shorter than that of control and DSF treated groups. However, no obvious histopathology with magnified resolution was found in seminal epithelial cells of all groups (Figure 3). This reduction of cell shape that was observed in the T2DM rats corroborated with significant decrease of their seminal epithelial height (*p* < 0.05, Figures 3 and 4). Significantly, administration of DSF extract (200 and 600 mg/kg BW) could improve the height of the epithelium in treated compared to untreated rats (*p* < 0.05, Figures 3 and 4).

### Effects of DSF extract on protein expressions in the seminal lysate of T2DM rats

**Figure 5.**
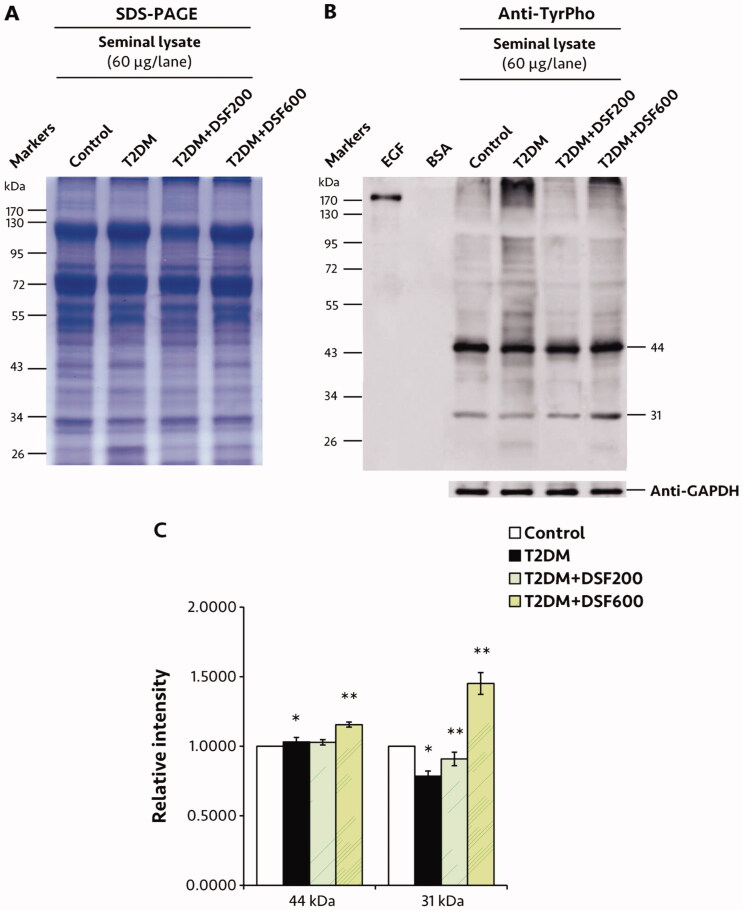
Representative protein profile based on SDS-PAGE pattern (A), western immunoblotting (B) and relative intensity of tyrosine-phosphorylated (TyrPho) protein expression (C) of the seminal lysate of control, T2DM, T2DM + DSF200 and T2DM + DSF600 rats. Protein expression in the control group was posited as 1, and that of the other groups is demonstrated relative to that of the control group. GAPDH was applied as an internal control. Values are expressed as mean ± SD. *Significantly different (*p*< 0.05) compared to the control group. **Significantly different (*p*< 0.05) compared to the T2DM group.

**Figure 6.**
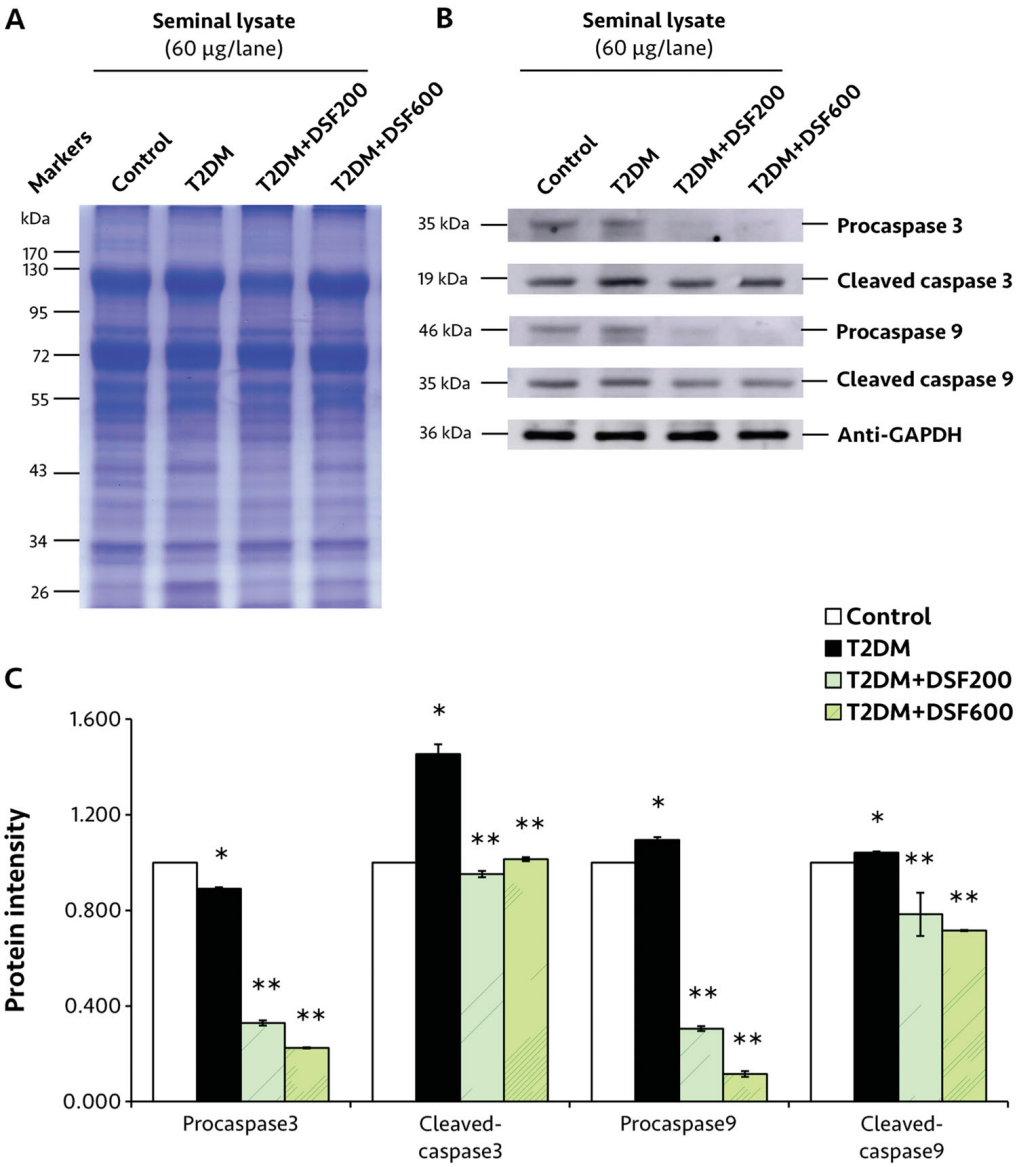
Representative protein profile based on SDS-PAGE pattern (A), western immunoblotting (B) and relative intensity of caspase 3 and 9 protein expressions (C) of the seminal lysate of the control, T2DM, T2DM + DSF200 and T2DM + DSF600 groups. Protein expression in the control group was posited as 1, and that of each of the other groups is demonstrated relative to that of the control group. GAPDH was applied as an internal control. Values are shown as mean ± SD. *Significantly different (*p*< 0.05) compared to the control group. **Significantly different (*p*< 0.05) compared to the T2DM group.

The pattern expression of TyrPho protein was differed at two major bands (44 and 31 kDa, Figure 5(B)). In the T2DM group, TyrPho protein expression at 44 kDa was significantly higher, while that at 31 kDa was significantly lower compared to the control group (*p* < 0.05, Figure 5(C)). DSF treatment resulted in significantly improved expression compared to that in untreated T2DM rats (*p* < 0.05, Figure 5(C)). In addition, western blot analysis revealed significant increase of pro and cleaved forms of caspase 3 and 9 expressions in the T2DM groups (*p* < 0.05, Figure 6(B,C)). Significantly, DSF extract in both groups could decrease the expressions of two apoptotic protein forms as compared to those in T2DM rats (*p* < 0.05, Figure 6(B,C)).

## Discussion

This study was the first to show alteration of seminal substances and proteins in T2DM rats. DM also affected the microstructure of the seminal epithelium, which supports the findings of a previous investigation (Tsounapi et al. 2017). Consistent with other studies, we found that DM significantly decreased the weight and increased histological abnormalities of seminal vesicles by reducing epithelial height and causing atrophy in animal models (Tsounapi et al. 2017; Yannasithinon and Iamsaard 2019). Treatment with DSF, which contains antioxidants and terpenoids (Chaimontri et al. 2021), reduces the occurrence of adverse features in the seminal vesicles in a way similar to that of synthetic antioxidant administration (Tsounapi et al. 2017). Hyperglycaemia induces oxidative stress resulting in diabetic complications (Oyenihi et al. 2015). Increased oxidative stress can lead to the aldehydic metabolism of lipid peroxidation, generating MDA molecules (Gaweł et al. 2004), elevated levels of which in the seminal plasma cause sperm might damage after ejaculation (Abdallah et al. 2009). Antioxidant supplements have been shown to correct MDA levels in the seminal vesicle plasma and tissue of DM animals (Tsounapi et al. 2017), which is similar to our findings. Likewise, protocatechuic acid (PCA) treatment has been shown to alleviate high MDA levels in the hippocampus and prefrontal cortex of chronic intermittent hypoxia (CIH)-induced rats (Yin et al. 2015). This may be due to PCA activity, as DSF has been shown to contain several antioxidants, particularly PCA, rengyolone and hallerone (Phanthong et al. 2015; Chaimontri et al. 2021). Similar to early reports (Tsounapi et al. 2017; Yannasithinon et al. 2021), serum testosterone was remarkably lower in the DM rats, a deficiency that improved with administration of DSF extract (Table 5). PCA has been shown to increase serum testosterone levels (Beytur et al. 2012; Phanthong et al. 2015; Adedara et al. 2018). Furthermore, DSF extract with water contains phytoandrogens (terpenoids), which might improve low serum testosterone (Chaimontri et al. 2021). Seminal vesicle function, including fluid secretion is regulated by the intracellular androgen receptor (AR) pathway. In this study, the phytoandrogens in DSF may improve biochemical parameters of the seminal fluid of T2DM rats via the AR pathway. As above mentioned, enhancement of seminal vesicular function and structure might result from DSF extract’s antioxidant activity causing improvement to serum testosterone and reduction of seminal MDA.

A previous report demonstrated that decreased weight and atrophy of seminal vesicle cause reductions in seminal fluid volume with the lower biochemical elements (Tsounapi et al. 2017). In terms of seminal biochemical components, ALP is involved in the production of fructose, a main energy source for sperm viability and movement (Rodríguez et al. 2013). In astheno-, oligo- and azoospermic men, low phosphorus (P) levels are associated with reduced fructose levels (Adamopoulos and Deliyiannis 2009), which is assumed to impair sperm energy. Additionally, the levels of Ca, MG and P were found to be lower in subfertile patients than those with normal fertility (Adamopoulos and Deliyiannis 2009). Assessment of MG and P levels in seminal plasma could determine the physiological function of the male reproductive tracts and accessory glands, especially the seminal vesicles (Adamopoulos and Deliyiannis 2009; Mahsud et al. 2013). Furthermore, the seminal plasma of infertile men also exhibits significantly lower FRA levels compared to that of their healthy counterparts (Tomaszewski et al. 1992). A previous study by Moreira et al. (2019) describes a correlation between of high FRA concentration and normal sperm morphology. Therefore, it is possible that the elevated FRA levels caused by DSF administration in our study may facilitate and maintain normal sperm morphology after ejaculation in T2DM. In addition, GOT and GPT have important roles in the metabolism of free radicals and protection the oxidative stress to the spermatozoa they cause (Talluri et al. 2017). In this study, DSF treatment in T2DM rats resulted in improvement of P, FRA, MG, GPT, GOT and ALP levels. It is possible that alteration of those components results from the oxidative stress scavenging ability of the antioxidants contained in DSF (Chaimontri et al. 2021). A previous report found that improvement of seminal parameter qualities in T2DM rats administered with DSF leads to improvement in overall semen quality (Yannasithinon et al. 2021).

The various proteins present in seminal plasma are thought to have important roles in sperm capacitation and the natural fertilization process in the female genital tract (Wang et al. 2020). Interestingly, TyrPho proteins are mainly localized in the testes, particularly in Sertoli and late spermatids, as described by Arad-Dann et al. (1993). They are further assumed to play essential roles in the spermatogenesis process and androgen synthesis (Iamsaard et al. 2014; Sampannang et al. 2017, 2018, 2019; Yannasithinon et al. 2021). A recent investigation demonstrated differences in 72 kDa TyrPho protein expression in the seminal lysate of T1DM and T2DM animals (Yannasithinon and Iamsaard 2019). The researchers postulated that that TyrPho protein might be a 72 kDa osteopontin, playing a role in early fertilization and embryonic development. Our study found that the expression of 44 and 31 kDa seminal TyrPho proteins was lower in T2DM rats; however, this was ameliorated by DSF administration. A previous study showed that 55, 48, 33, 31 and 18 kDa seminal proteins are members of the heparin-binding protein (HBP) family (Patel et al. 2016; Singh et al. 2016). HBP, a major seminal protein, has been postulated as a biochemical marker to predict semen quality (Manjunath et al. 2002; Patel et al. 2016; Singh et al. 2016). It is possible that both 44 and 31 kDa might be HBP, because it is a prominent seminal protein secreted from the accessory sex organs as previously demonstrated (Patel et al. 2016; Singh et al. 2016). We might conclude that increasing the expression of 44 and 31 kDa seminal proteins might improve sperm quality and male sub/infertility in DSF-treated T2DM rats.

Moreover, a previous study showed strong positive staining for cleaved caspase-3 (apoptosis marker) on the seminal tissue of DM rats by immunohistochemistry (Tsounapi et al. 2017). Indeed, the reduced androgen levels in castrated rats were shown to upregulate apoptotic pathway in seminal epithelial cells (Tanji et al. 2003). Interestingly, antioxidant treatment in DM- or CIH-induced rats has been shown to diminish the expression of caspase 3 in seminal and brain tissue and caspase 9 mRNA in testicular tissue (Yin et al. 2015; Tsounapi et al. 2017; Nna et al. 2019). We observed decreased expression of seminal caspase 3 and 9 in T2DM animals after DSF extract treatment. The antioxidants in DSF might upregulate MDA levels, resulting in decreased caspase 3 and 9 expression. Moreover, a previous study demonstrated that DSF reduced MDA levels in T2DM testes (Yannasithinon et al. 2021). However, its caspase expression was investigated (Yannasithinon et al. 2021). In contrast, our study showed that decreased MDA levels directly correlated with reductions in caspase expression in T2DM seminal vesicles treated with DSF. In addition, we hypothesize that the antioxidant capacity of DSF might result in scavenging of excess oxidative stress, ameliorating apoptosis and atrophy of T2DM seminal vesicles.

## Conclusions

*Dolichandrone serrulata* flower extract ameliorated the destructive functions and structures of T2DM seminal vesicles by decreasing MDA level and TyrPho and caspase (3 and 9) protein expression and increasing levels of inorganic biochemical substances and serum testosterone. This finding might aid in the development of a DSF extract as a food supplement to reduce progressive T2DM complications in the reproductive organs, especially the male seminal vesicles, which may improve seminal quality as well.
